# Autophagy Inhibition Contributes to the Synergistic Interaction between EGCG and Doxorubicin to Kill the Hepatoma Hep3B Cells

**DOI:** 10.1371/journal.pone.0085771

**Published:** 2014-01-21

**Authors:** Li Chen, Hui-Lan Ye, Guo Zhang, Wen-Min Yao, Xing-Zhou Chen, Fa-Can Zhang, Gang Liang

**Affiliations:** 1 New Drug Research & Development Center, The First Affiliated Hospital of Guangxi Medical University, Nanning, Guangxi, P. R. China; 2 Department of Gastroenterology, The People's Hospital of Guangxi Zhuang Autonomous Region, Nanning, Guangxi, P. R. China; 3 Pharmacy School of Guangxi Medical University, Nanning, Guangxi, P. R. China; Taipei Medical University, Taiwan

## Abstract

(-)-Epigallocatechin-3-*O*-gallate(EGCG), the highest catechins from green tea, has promisingly been found to sensitize the efficacy of several chemotherapy agents like doxorubicin (DOX) in hepatocellular carcinoma (HCC) treatment. However, the detailed mechanisms by which EGCG augments the chemotherapeutic efficacy remain unclear. Herein, this study was designed to determine the synergistic impacts of EGCG and DOX on hepatoma cells and particularly to reveal whether the autophagic flux is involved in this combination strategy for the HCC. Electron microscopy and fluorescent microscopy confirmed that DOX significantly increased autophagic vesicles in hepatoma Hep3B cells. Western blot and trypan blue assay showed that the increasing autophagy flux by DOX impaired about 45% of DOX-induced cell death in these cells. Conversely, both qRT-PCR and western blotting showed that EGCG played dose-dependently inhibitory role in autophagy signaling, and that markedly promoted cellular growth inhibition. Amazingly, the combined treatment caused a synergistic effect with 40 to 60% increment on cell death and about 45% augmentation on apoptosis versus monotherapy pattern. The DOX-induced autophagy was abolished by this combination therapy. Rapamycin, an autophagic agonist, substantially impaired the anticancer effect of either DOX or combination with EGCG treatment. On the other hand, using small interference RNA targeting chloroquine autophagy-related gene Atg5 and beclin1 to inhibit autophagy signal, hepatoma cell death was dramatically enhanced. Furthermore, in the established subcutaneous Hep3B cells xenograft tumor model, about 25% reduction in tumor growth as well as 50% increment of apoptotic cells were found in combination therapy compared with DOX alone. In addition, immunohistochemistry analysis indicated that the suppressed tendency of autophagic hallmark microtubule-associated protein light chain 3 (LC3) expressions was consistent with thus combined usage *in vitro*. Taken together, the current study suggested that EGCG emerges as a chemotherapeutic augmenter and synergistically enhances DOX anticancer effects involving autophagy inhibition in HCC.

## Introduction

Hepatocellular carcinoma (HCC) is one of the most prevalent cancers worldwide and is accounting for 85 to 90% of all primary liver cancer which represents approximately 4% of all new cancer cases diagnosed [1]. Although liver transplantation is considered the most effective method for advanced HCC, surgical resection is used as upfront treatment due to the living donor problems [2] and it is still applicable to only a small proportion of patients with high recurrence rate about 50% at 2 years and 75% at 5 years after the resection, respectively [3]. To this day, diversified nonsurgical managements such as bland particle embolization, chemoembolization, radioembolization, stereotactic body radiation therapy and traditional chemotherapy have been importantly applied to patients with HCC. However, since their toxic side effects and most common multiple drug resistance [4], [5], the overall prognosis still remains frustratingly poor.

Among those valuable cytostatic agents available in the chemotherapeutic guideline, DOX was routinely used as a single drug for treatment of patients with HCC [6]. Indeed, DOX-induced myocardial toxicity and related resistance in tumor cells are really serious, especially with a low response rate of about 15 to 20% in HCC [6]. In this regards, a couple of combination regimens were suggested for DOX usage to deal with the controversy between its potency and side-effect. Our previous study has shown that EGCG severed as a promising chemosensitizing enhancer for DOX in HCC treatment [7]. However, the detailed mechanism responsible for this combined strategy towards tumor is not known with certainty. Recently, as soon as a novel mechanism of autophagy underlying the carcinogenesis has been reported, it raises a promising attempt to figure out whether the interaction between DOX and EGCG involved autophagic flux during the therapeutic process for HCC.

Autophagy is an evolutionary conserved cellular process which degrades and recycles intracellular constituents. As it is activated upon various stressful stimuli including physiological disturbance and pathological conditions, the modulation of autophagy is a new pattern to determine cell fate and provides mechanistic insight into pathogenesis and therapy of varies diseases in mammalian [8], [9]. However, how does autophagy plays a role in chemotherapy for malignant tumor remains controversial. Recent study identifying the autophagy-mediated necroptosis mechanism using a novel chalcone derivative chalcone-24 established the role of autophagy for overcoming chemoresistance [10]. Additionally, another study in breast cancer cells revealed that ceramide transporter depletion promoted sensitization to diverse cytotoxics, which was mediated by enhanced autophagy flux [11]. These evidences indicated that autophagy induction may contribute to the efficacy of some anticancer agents. Conversely, the overwhelming majority of studies supported that autophagy inhibition significantly increased cell death in gastrointestinal and hepatic cancer in response to various anticancer agents [12]–[19]. For instance, multi-kinase inhibitors such as sorafenib stimulated autophagy in hepatoma cells which attenuated its anti-cancer effects [20]. Therefore, to appropriately modify autophagy signals would be worth investigating to clarify the mechanism underlying the combined strategy for HCC.

EGCG as the highest catechins content in green tea has a variety of physiological and pharmacological activities. It has been shown that EGCG not only induced the apoptosis and inhibited the proliferation in tumor cells [21], [22], but also increased sensitivity to traditional antineoplastic drugs and reverse multidrug resistance in hepatoma cells [7]. Recently, it has been shown that EGCG exerted these beneficial effects involving autophagy flux regulation and the specific mechanism differs from different cells and diseases [23]–[26]. However, whether the regulation of autophagic signaling is responsible for its anticancer augmentative effect in HCC is still not known. In this study, we aimed to determine the synergistically antitumor effect mediated by autophagy signals in response to EGCG cooperated with DOX in HCC and identify a new autophagy inhibitor as a potential augmenter for cancer therapy.

## Materials and Methods

### Reagents

EGCG, 3(4,5-demethylthiazole-2-yl)-2,5-diphenyl tetrazolium-bromide (MTT) were purchased from Sigma. Doxorubicin was purchased from Hisun Pharmaceutical Co. Ltd. (Zhejiang, China). Human anti-beclin1 polyclonal antibody was from Cell signaling Technology. Anti-LC3 and anti-Atg5 polyclonal antibody were purchased from Novus Biologicals. Human anti-GAPDH polyclonal antibody and all horseradish peroxidase–conjugated secondary antibodies were purchased from Santa Cruz Biotechnology. Trizol was from Invitrogen. cDNA reverse transcription system, SYBR® Premix Ex Taq™ II reagent kits were from Takara Biotechnology (Dalian, China). PCR primers were synthesized by Sangon Biotechnology (Shanghai, China). Terminal deoxynucleotidyl transferase dUTP nick end labeling (TUNEL) kits and enhanced chemiluminescence were purchased from Merck. High glucose dulbecco's modified eagle's medium and fetal bovine serum were from Thermo. Trypsin was purchased from Amresco. Polymer Detection System Kits for immunohistological staining was from ZSGB-Bio (Beijing, China). Horseradish Peroxidase Color Development Kits was purchased from Beyotime Biotechnology.

### Cell lines and animals

The human HCC cell lines Hep3B cells (Cell Resource Center of Shanghai Institute for Biological Sciences) were maintained in high-glucose dulbecco's modified eagle's medium supplemented with 10% heat-inactivated fetal bovine serum and 100 units/ml penicillin-streptomycin (Solarbio Science and Technology, Beijing, China) at 37°C in a humidified incubator in 5% [v/v] CO_2_ atmosphere. Cells in logarithm growth stage were plated in plates or covered culture dishes at different densities for the following designed studies. Male nude mice (four to six weeks old, Animal Research Center of Guangxi Medical University) were maintained under specific pathogen free (SPF) conditions.

### MTT assay

Cells were plated at a density of 7000 per well of a 96-well plate and, 24 hours after plating, treated as the indicated concentrations. Twenty microliters MTT with a concentration of 5 mg/ml was added to each well for an additional 4 hours. The blue MTT formazan precipitate was then dissolved in 150 µL of dimethyl sulfoxide per well with incubation for 10 minutes in a rotary platform at 37°C. Cell proliferation inhibition ratio was calculated according to the absorbance at a wavelength of 490 nm (A value) in each well by ELISA analyzer (GF-M3000,ShangDong province, China). Cell proliferation inhibition ratio (%) = (A value of control group–A value of treated group)/A value of control group ×100%.

### Analysis of in vitro drug interaction

As previous studies [27], [28], cells were seeded at a density of 7000 per well of a 96-well plate and, 48 hours after cells attached, treated as the indicated agents and concentrations. MTT assay was used to detect the absorbance at a wavelength of 490 nm as the OD value. The coefficient of drug interaction (CDI) is calculated as follows: CDI = AB/(A×B). According to the absorbance of each group, AB is the ratio of the combination groups to control group; A or B is the ratio of the single agent group to control group. Thus, CDI value <1,  = 1 or >1 indicates that the drugs are synergistic, additive or antagonistic, respectively.

### Trypan blue assay

Cells were plated at a density of 2×10^5^ per well of a 12 well plate. At the end of treatment as described as the figure legend, cells in each well were calculated under a light microscope (Olympus, CKX-41).

### Annexin V/propidium iodide assay

Cells were plated at a density of 5×10^5^ per well of 6 well plates. After treatment as indicated as the Figure legend, apoptotic cells were evaluated *in vitro* by Annexin V/propidium iodide staining (BD PharMingen) according to the manufacturer's instructions and then were analyzed using flow cytometry (Mansfield, MA).

### Transfection of cells with small interference RNA (siRNAs)

Cells were plated at a density of 1×10^5^ per well of 12 well plates in antibiotic-free medium. After plating 24 hours, cells were 30–50% confluent and then infected with a variety of constructs. Two microliter Lipofectamine™ 2000 (Invitrogen) and 50 nM siRNA (scrambled or experimental) were diluted into 100 µL of Opti-MEM® I Reduced Serum Medium (Gibco), respectively (one portion for each sample). Diluted siRNA was added to the diluted Lipofectamine 2000 for each sample and incubated at room temperature for 25 minutes. The mixture was added to each well of cells containing 800 µL antibiotic-free medium for a total volume of 1000 µL medium. An equal volume of medium was replaced after 4 hours incubation. Cells were incubated for 24 hours, then treated with the indicated concentrations of DOX, EGCG and a combined pattern, and subsequently analyzed after 24 hours treatment.

### Electron microscopy

Hep3B cells were incubated with doxorubicin of 2.5 µM for 24 hours, and then collected in eppendorf tubes by centrifugation after being digested with 0.25% trypsin. Prior to embedding, cells were fixed with 4% glutaraldehyde and 1% osmium tetraoxide overnight. Then cells were embedded in epoxide resin, followed by ultrathin sections (100 nm) prepared on an ultramicrotome and next, were double stained with uranyl acetate and lead citrate. Images of the autophagy in cytoplasm were viewed with a transmission electron microscope (Hitachi H7650, Japan).

### Fluorescence microscopy

Cells were plated in 24-well plates with 5×10^4^ cells per well and treated with indicated agents and indicated time. Briefly, cells were fixed with 4% paraformaldehyde for 10 minutes. Subsequently, the cells were permeabilized with 0.5% Triton X-100 for 15 minutes, washed with PBS and blocked with 1% BSA for 30 minutes at room temperature. The cells were treated with LC3 antibody diluted by 1% BSA and incubated overnight. Prior to staining with 5 ug/ml 4′,6-diamidino-2-phenylindole (SIGMA), cells were incubated with secondary antibody conjugated with fluorescein isothicyanate for 1 h. The slices were examined under the fluorescent microscope (Olympus, IX-71).

### Western blotting

Total protein was extracted from cells or tumor tissues and lysed in RIPA lysate [20 mM Tris-HCl (pH = 8.0), 1%NP40, 150 mM NaCl, 2 mM ethylene diamine tetraacetie acid (PH = 8.0), 0.1%SDS] which contained protease inhibitor (Roche Molecular Biochemicals). Equal amounts of proteins were loaded onto 10% SDS-PAGE and transferred to a polyvinylidene difluoride membrane (Millipore) which subsequently was blocked with 5% nonfat milk and incubated in corresponding primary and secondary antibodies as designed. All immunoblots were visualized by electronic chemiluminescence (PerkinElmer) according to the manufacturer's instructions and then digitally scanned. The density of protein band presenting the protein expression level was analyzed using Image J software (http://rsb.info.nih.gov/ij/).

### Quantatative real-time quantitative polymerase chain reaction (qRT-PCR)

At the end of treatments, cells interfered with drugs or infected with siRNAs were rinsed with cold PBS solution. Trizol reagent (Invitrogen) was used for RNA extraction, followed by the measurement of RNA concentration. The reverse transcription of cDNA was processed using a SYBR® Premix Ex Taq™ II reagent kits. SYBR method was used to detect the expression of autophagy related gene Atg5 and beclin1. The synthesized primers were as follows: Atg5 forward primer, 5′-CCAAAGCAGCATTGATGACCA-3′; Atg5 reverse primer, 5′-AGCCACAGGACGAAACAGCTT-3′. beclin1 forward primer, 5′-ACAGTGGACAGTTTGGCACA-3′; beclin1 reverse primer, 5′-CGGCAGCTCCTTAGATTTGT-3′. Specific primers for the GAPDH were used as control and the primers were forward primer, 5′-CATGAGAAGTATGACAACAGCCT-3′ and reverse primer, 5′-GTCCTTCCACGATACCAAAGT-3′. PCR conditions were one cycle of 95°C for 5 seconds (predenaturation) and forty cycles of 95°C for 5 seconds (denaturation) and 60°C for 34 seconds (renaturation). The relative expression levels of mRNA were determined by the formula, 2^−ΔΔCt^.

### Establishment of supcutaneous xenograft tumor model in nude mice

The nude mice were subcutaneously inoculated with cell suspension containing 1×10^7^ Hep3B cells per mouse in the right side fossa axillaries. The subcutaneous tumors were monitored and when the volume of tumor reached 100 mm^3^, the nude mice were subjected to medical intervention. Based on the weight and the volume of the tumor, the mice were randomly divided into 4 groups with 6 in each, including the control group, the EGCG group (50 mg/kg, qd, ig; EGCG), DOX group(2 mg/kg, q4d, ip; DOX) and DOX combined with EGCG (EGCG+DOX group). The tumor weight was measured every 2 days and the tumor size was monitored every 4 days by vernier caliper. Measured values were used to calculate the tumor volume according to the formula [length (mm)×width (mm)^2^]/2. Fifteen days after treatment, all rats were humanely sacrificed to dissect and weight the tumor tissues. A portion of the tumor tissue was fixed in 10% formalin for subsequent histological analysis, and the remaining tissue was stored at −80°C for molecular studies. All experimental procedures were approved by the Animal Ethics Committee of the First Affiliated Hospital of Guangxi Medical University and the People's Hospital of Guangxi Zhuang Autonomous Region, Nanning, China.

### Immunohistochemistry

Tumor tissues from control group and treatment groups were used for immunohistochemistry following the Polymer Detection System Kits. Sections (4 µm thick) from paraffin-embedded tumors were deparaffinized and then rehydrated using xylene and ethanol, and next, immersed in 3% hydrogen peroxide solution for 10 min in dark to block endogenous peroxidases. After rinsed with double-distilled water and immersed in PBS solution for 3 times, sections were boiled for 10 minutes in 10 mM citrate buffer solution (pH 6.0) for antigen retrieval. Slides were incubated overnight at 4°C with anti-LC3 (1∶100). The appropriate peroxidase-conjugated secondary antibody was added to specimens and incubated for 30 minutes at 37°C. Visualization was performed using the DAB Kits (ZSGB-BIO, China) following the manufacturer's instructions. All slides were counterstained with hematoxylin and eosin (HE).

### HE staining

The deparaffinization and rehydration were conducted as described above. Prior to dehydration, nuclei were stained with hematoxylin and cytoplasm with eosin. Mountant was dropped on the slide and a cover glass was put on it. Morphological changes were obtained under the light microscope.

### TUNEL assay

The slides were next deparaffinized and rehydrated and treated with proteinase K for 20 minutes. The tissue sections were then analyzed with FragEL™ DNA Fragmentation Detection Kits (Merck, America) followed the manufacturer's instructions and visualized using fluorescence microscope. The percentage of TUNEL-positive cells was calculated by dividing the number of TUNEL-positive cells by the number of 4′,6-diamidino-2-phenylindole-positive nuclei at high magnification for three fields in each tumor sample.

### Statistical analysis

Statistical analysis was performed using SPSS13.0 software. Experiments were repeated at least three times with consistent results. Quantitative data were presented as mean ± SE. Comparison of the effects of various treatments was performed using One-Way ANOVA analysis and Pearson's correlation. Tumor mean diameter, and mean volume were analyzed for statistical significance using paired Student's t tests. *P*<0.05 was considered statistically significant.

## Results

### Autophagy was observed in Hep3B cells and up-regulated by DOX administration

**Figure 1:**
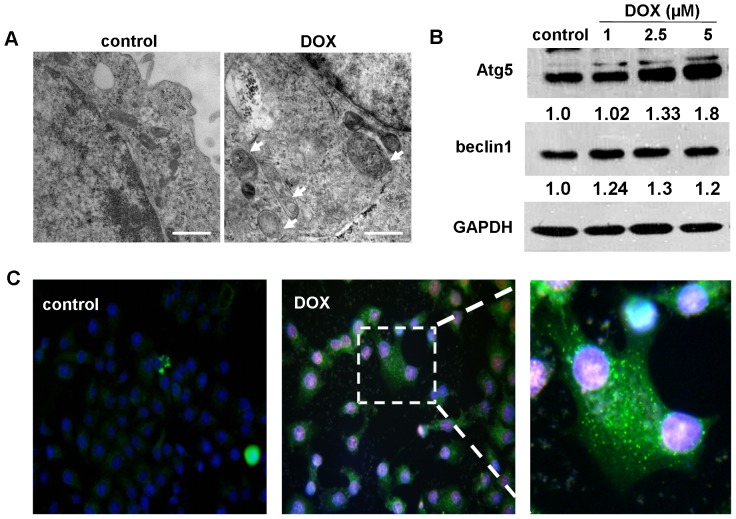
DOX treatment was found to increase autophagic activity in Hep3B cells. (A) Electron microscopic technology showed up-regulated numbers of autophagosomes in Hep3B cells treated with 2.5 µM DOX versus control. Arrows indicate autophagic structures. Scale bars represent 500 nm. Magnification, ×40000. (B) Immunoblots showed increased expression levels of Atg5 and beclin1 in Hep3B cells treated with DOX of 1, 2.5 and 5 µM for 24 h compared with control. Protein ratios normalized to GAPDH were used to quantify fold change relative to control and are shown below each blot. Data are from a representative study (n = 3). (C) Immunofluorescence analysis indicated that elevated LC3 fluorescent signals were visualized in cells administrated with 2.5 µM DOX.

To confirm whether autophagic activity is altered with DOX treatment, transmission electron microscopy was done to visualize autophagosomes in the cytoplasm in Hep3B cells. Typical autophagosome was defined as a double-membraned structure containing intracellular organelles and cytoplasmic contents such as mitochondria, endoplasmic reticulum and ribosome [29]. As showed in Fig. 1A, there was a marked increment in the number of autophagic structures in cells treated with DOX (2.5 µM) (pannel right) compared with the vehicle (control) (pannel left). Simultaneously, western blotting showed that the DOX dose-dependently induced increasing expression levels of autophagic protein Atg5 and the expression of beclin1 also mildly elevated in these cells. (Fig. 1B). Moreover, by immunofluorescence microscopy, autophagic activity with LC3 labeled in cultured Hep3B cells was measured. It was found that there was an evidently increased green punctae in cells treated with DOX versus vehicle (Fig. 1C). These results were consistent with the previous study [30].

### DOX-induced antitumor effects were enhanced by autophagy inhibition

**Figure 2:**
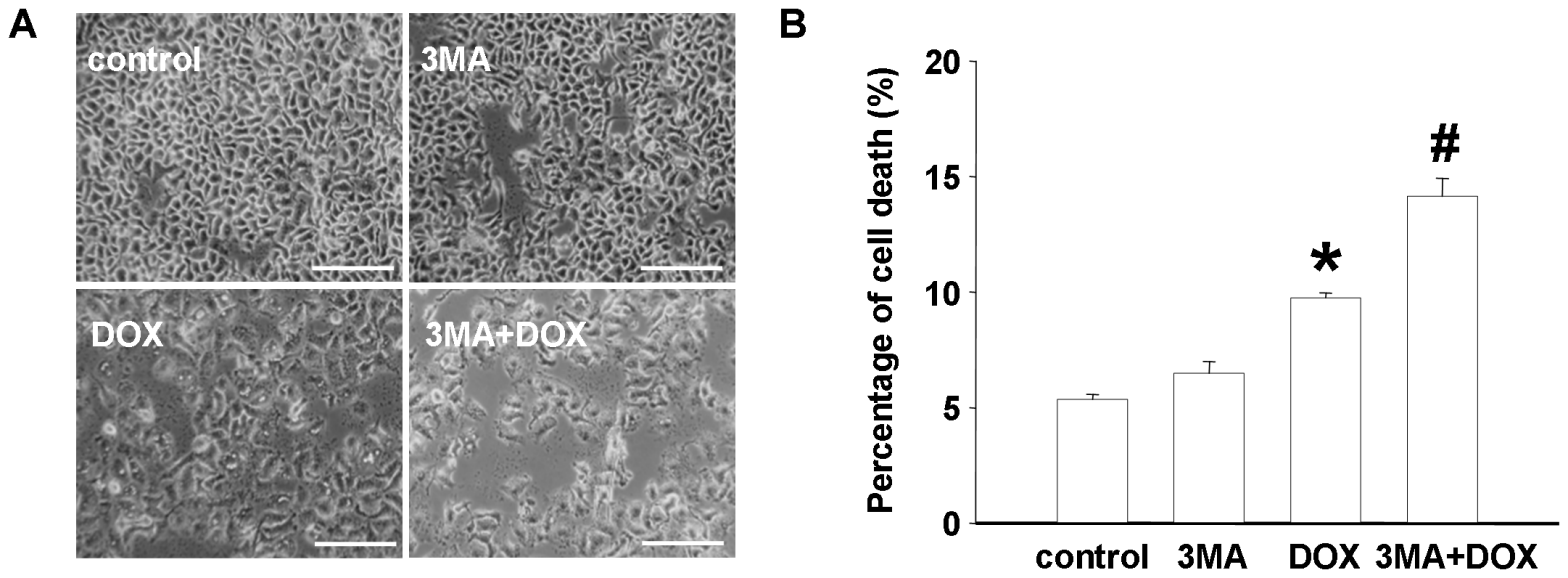
Autophagy suppression enhanced DOX-induced growth inhibition and cell death of Hep3B cells. Hep3B cells were treated with vehicle (control), 2 mM 3MA, 2.5 µM DOX, or both 3MA and DOX for 24 h. Light microscopic images recorded the morphology (A) and trypan blue assay determined the cell death (B); Columns, percentage of trypan blue-positive cells; bars, SE. Data was from a representative of three independent studies. Bars = 200 µm. *p<0.05 *vs*. control, ^#^p<0.01 *vs*. control.

Under the light microscope, administration of DOX was found to exert substantial antiproliferative effects on hepatoma Hep3B cells. Interestingly, while pre-treated those cells with three-methyladenine (3MA), a representative autophagic antagonist, the DOX-induced proliferative inhibition effects was dramatically aggravated. The trypan blue assay further confirmed autophagic suppression amplified DOX-induced cell death with an enhancement of about 45% (Fig. 2).

### EGCG significantly inhibited autophagic activity and suppressed proliferation in vitro

**Figure 3:**
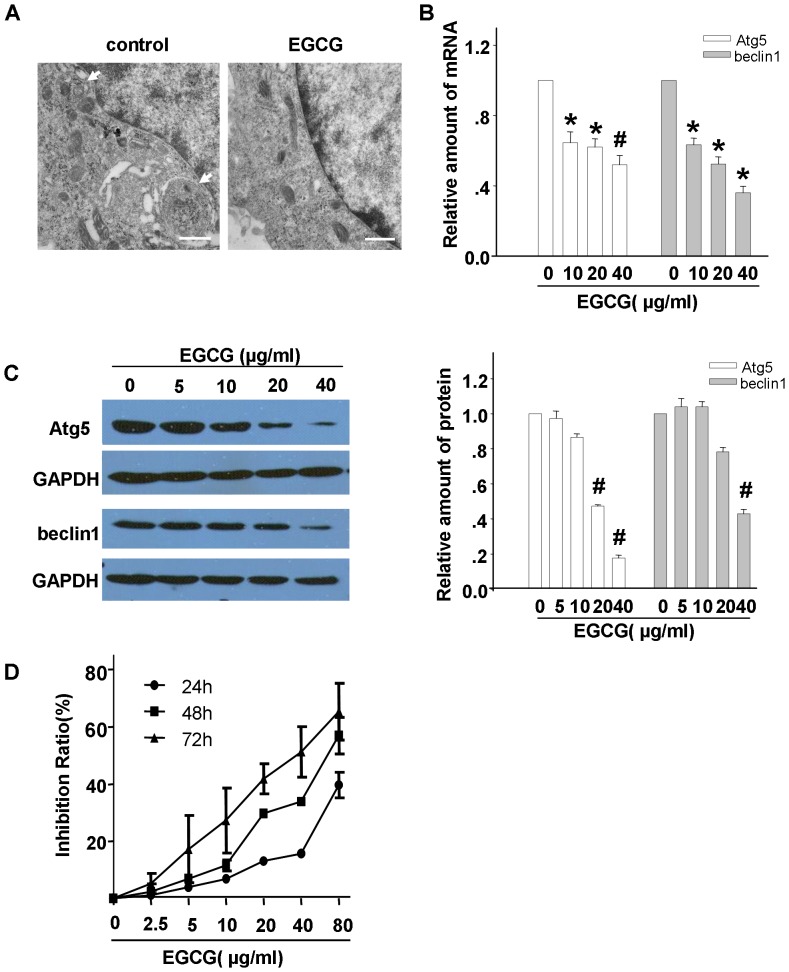
Dose-dependent inhibited effect of EGCG on the autophagy and proliferation in Hep3B cells. (A) EGCG (40 µg/ml) was found to reduce the autophagosome number in Hep3B cells. Arrows indicate autophagic structures. (B) Exposed to EGCG of 10, 20, 40 µg/ml for 24 h, the expression levels of Atg5 and beclin1 in Hep3B cells were determined at the RNA levels by qRT-PCR. (C) Cell lysates following treatment with varies concentrations of EGCG were subjected to western blotting. Protein ratios normalized to GAPDH were used to quantify fold change relative to control. Results shown are representative of three independent experiments and error bars indicate SE. (D) EGCG exerted inhibition effects on proliferation of Hep3B cells in dose- and time-dependent manner after EGCG treatment with the indicated concentrations. Bars = 500 nm. Magnification, ×40000. *p<0.05 *vs*. control, ^#^p<0.01 *vs*. control.

To address the prospect that EGCG regulates autophagic activity, electron microscopy showed a decrement of autophagic vacuoles in Hep3B cells treated with EGCG (Fig. 3A). To further confirm that autophagic activity was down-regulated by EGCG, a dramatic reduction of mRNA expression level of autophagic genes was reflected by qRT-PCR, which behaved as a dose-dependent manner (Fig. 3B). Concurrently, western blotting indicated a decreased expression of Atg5 in cells treated with 20 µg/ml and 40 µg/ml EGCG of about 50% and 80%, respectively. Meanwhile, the suppressed expression levels of beclin1 protein of about 60% also detected in those cells treated with 40 µg/ml EGCG for 24 hours (p<0.01) (Fig. 3C). In addition to the autophagy inhibition effects, MTT assay showed that proliferation suppression on Hep3B cells was also found to exert in a dose- and time-dependent manner following EGCG treatment (Fig. 3D). An inverse correlation between the exposure concentration of EGCG and protein or gene expression of Atg5 and beclin1 was potently showed when Pearson's correlation was used, and a positive correlation was showed between EGCG concentrations and cell-growth inhibition ratio.

### Combination of EGCG and DOX synergistically facilitate antitumor effects in Hep3B cells, which involved autophagic regulation

**Figure 4:**
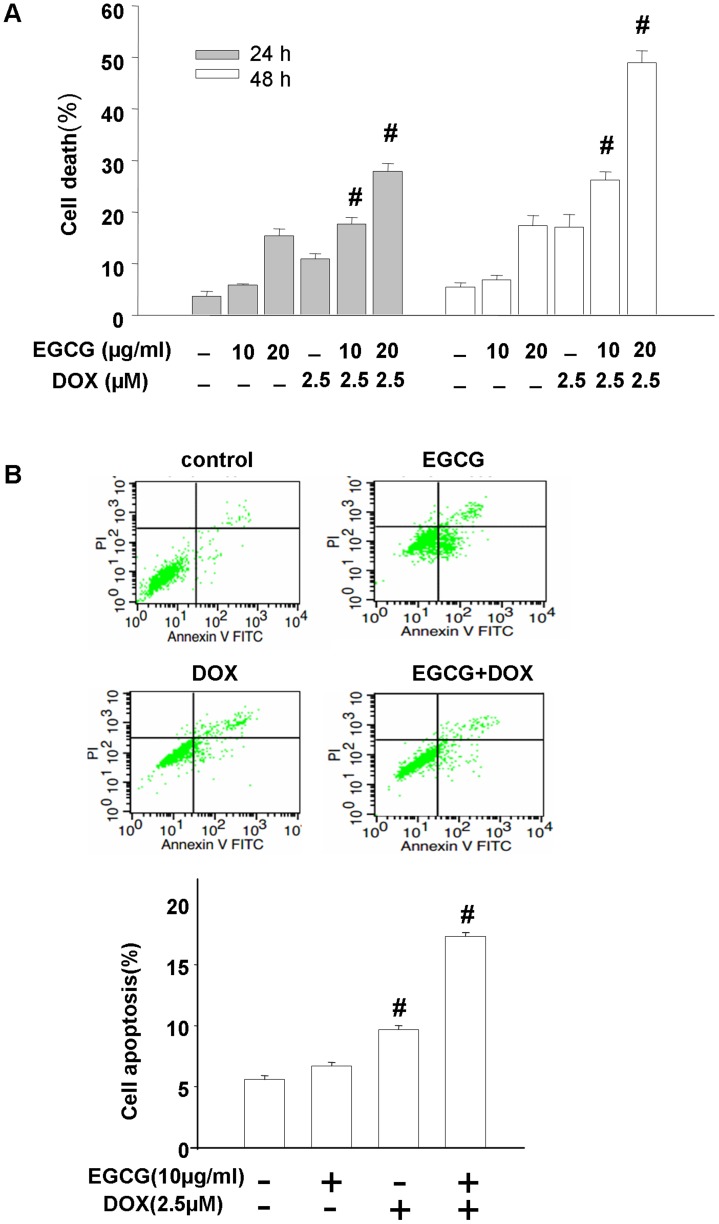
Combination of EGCG and DOX promoted cell death and apoptosis in Hep3B cells. (A) Trypan blue assay characterized the cell death of Hep3B cells treated with EGCG (10 µg/ml, 20 µg/ml) in the presence or absence of DOX (2.5 µM) for 24 h and 48 h. (B) Flow cytometry analyzed the apoptosis of Hep3B cells after addition of EGCG (10 µg/ml) in the presence or absence of DOX (2.5 µM) for 24 h. The lower panel is the summarized data. Results are representative of three independent experiments and error bars indicate SE. ^#^p<0.01 *vs*. control.

**Figure 5:**
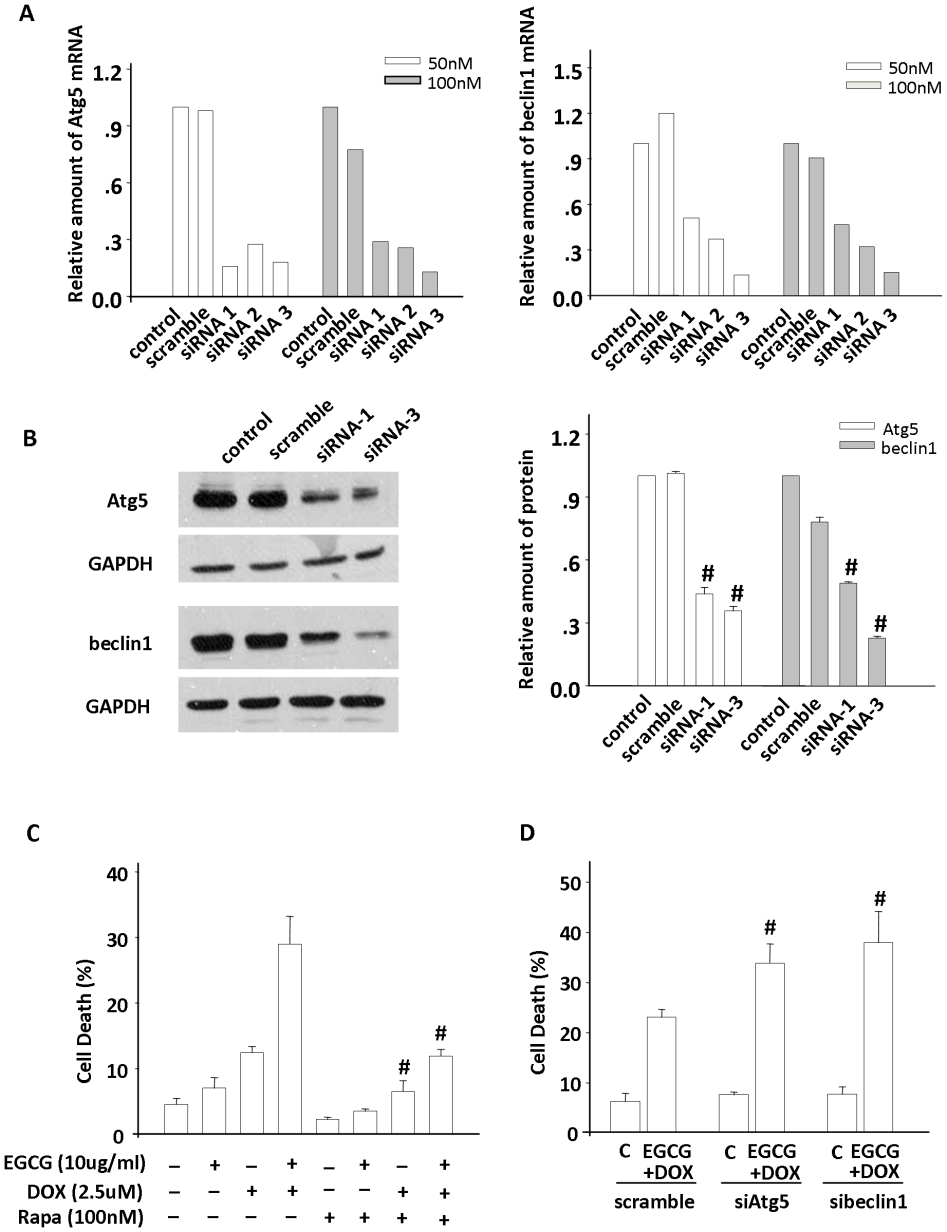
Combination effects of EGCG and DOX on Hep3B cells involved autophagic modulation. Genetic inhibition of autophagy in Hep3B was conducted with siRNAs targeting at Atg5 and beclin1. Effective knockdown of autophagy gene or protein expression levels with each siRNA was confirmed by qRT-PCR (A) and western blotting (B). By trypan blue staining, it was showed that (C) rapamycin (Rapa,100 nM), an agonist, substantially impaired the cell death in Hep3B treated with EGCG(10 µg/ml) and DOX(2.5 µM) and (D)blocking autophagy by siRNAs targeting at Atg5 and beclin1 enhanced cell death of these cells in the presence of EGCG and DOX for 48 h. Cumulative results from three independent experiments were shown as mean ± SE. C, control. ^#^p<0.01 *vs*. control.

Given the evidence provided above that the opposing actions towards autophagy activity as well as cell fate exerted by both ECCG and DOX treatment in Hep3B cells, we proceeded to define the interact effects of EGCG on DOX-induced antitumor effects. As expected, according to the MTT assay and the coefficient of drug interaction (CDI) shown in table 1, EGCG and DOX yielded synergistic interactions across a wide concentration range (CDI<1). In particular, a lowest CDI value (0.79±0.06) was presented in the combination of 10 µg/ml EGCG and 2.5 µM DOX. By trypan blue assay, synergistic inhibitory effects on the viability of Hep3B cells were also observed in these combined two compounds. For 24 h incubation, a significantly greater cell death percentage was showed in the 10 µg/ml EGCG co-treatment cells compared with the DOX-challenged cells (17.67±0.52% *vs*. 10.89±0.43%, p<0.01) and for 20 µg/ml EGCG co-treatment cells (27.86±0.64% *vs*. 10.89±0.43%, p<0.01). When the exposure time extended from 24 h to 48 h, a higher cell death percentage of 48.99±2.31% was presented in the combined pattern (Fig. 4A). As for the flow cytometry detection, co-treatment was found to increase the apoptosis by about 45% versus DOX-suffered cells (Fig. 4B), which was consistent with the trypan assay. These results demonstrated that EGCG, in combination with DOX, presented a significantly synergistic anticancer effect on Hep3B cells. Meanwhile, siRNA interference technique targeting at Atg5 and beclin1 genes was carried to suppress autophagy. Gene knockdown effect was validated by qRT-PCR and western blotting (Fig. 5A, B). Rapamycin, an inhibitor of mTOR, impaired the cell death of about 60% in the co-treatment Hep3B cells (Fig. 5C). However, it was refreshing that specifically inhibiting the autophagy pathway by siRNAs powered the cell death (Atg5 siRNA, beclin1 siRNA and scrambled siRNA; 33.84±3.84, 37.99±6.12 and 23.03±1.60, respectively) (Fig. 5D).

**Table 1:** Dosage inhibitory effects of both EGCG and DOX on the proliferation of Hep3B cells (n = 9).

EGCG(µg/ml)	DOX(µM)	Growth inhibitory effects (OD)			CDI
		EGCG	DOX	EGCG+DOX	
0	0	0.96±0.02	0.96±0.02	0.96±0.02	
2.5	0.725	0.92±0.01	0.85±0.00	0.72±0.01	0.88±0.03
5	1.25	0.86±0.01	0.76±0.02	0.61±0.03	0.89±0.09
10	2.5	0.83±0.01	0.73±0.02	0.50±0.03	0.79±0.06
20	5	0.75±0.00	0.50±0.04	0.36±0.03	0.91±0.07
40	10	0.40±0.03	0.43±0.43	0.16±0.01	0.88±0.06

Drug interaction was measured as described in materials and methods with increasing concentrations of EGCG, DOX or both agents for 48 h. CDI<1 indicates a synergistic effect, CDI = 1 indicates an additive effect, CDI>1 indicates an antagonistic effect.

### Anti-tumor activity of EGCG and DOX in vivo

**Figure 6:**
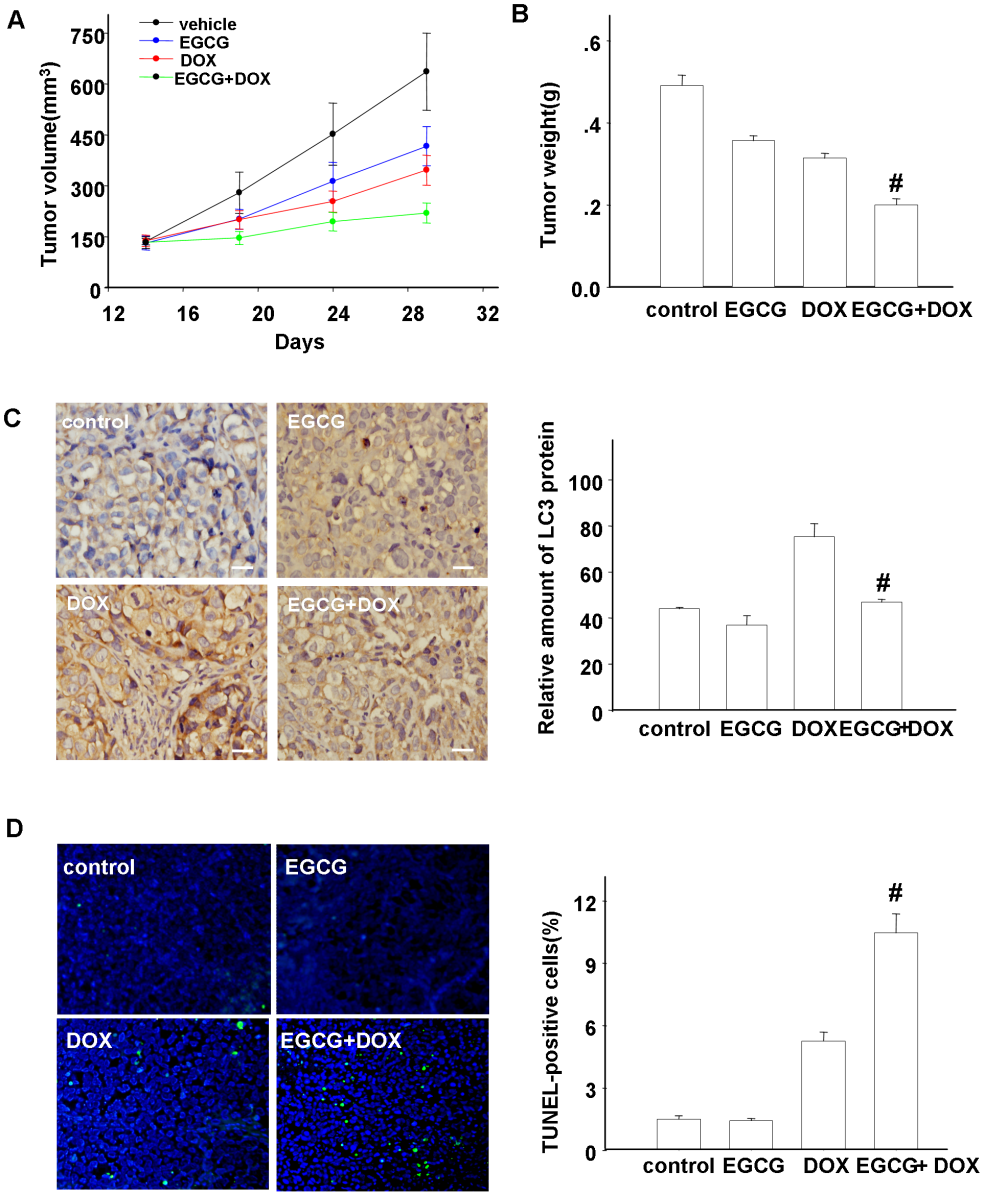
Contribution of autophagy and apoptosis to anti-tumor effects of EGCG and DOX in HCC model. Nude mice were subcutaneously injected with Hep3B cells. When the volume of tumor reached 100^3^, the mice were divided into corresponding treatment groups based on both volume and weight. Tumor volume was recorded every 4 days (A) and the tumor weight was recorded (B) when the tumors were excised after 15 days treatment. The data represents means and standard deviations and error bars indicate SE. (C) LC3 protein expression in each tumor tissue section was measured by immunochemistry. Magnification, 400×. (D) Apoptosis in each tumor tissue section was measured by TUNEL staining. Positive cells were determined in three independent experiments. Three random fields representing 200 tumor cells were counted. Magnification, 200×. Cumulative results were shown as mean ± SE and error bars indicate SE. Bars = 20 µm. *p<0.05 *vs*. control, ^#^p<0.01 *vs*. control, n = 6.

The size and weight of the tumors in the EGCG or DOX treatment groups were slightly diminished compared with the control group. But for the combination treatment group, a marked inhibition of tumor size and weight presented in comparison with DOX-treated tumors (219.66±12.15 mm^3^ *vs*. 346.35±17.98 mm^3^, P<0.01; 0.20±0.01 g *vs*. 0.31±0.01 g, P<0.01) (Fig. 6 A, B). The harvested tumor tissues were subjected to HE staining, immunohistochemistry, TUNEL assay and western blotting. HE staining showed the structure of the tumor tissues presented as tumor nodules, cells organized in disorder, nuclei of different sizes with pathological karyokinesis. Visible necrosis and abundant lymphocytes and monocytes were evident in DOX treatment group (Figure not shown). Immunohistochemistry showed decreased expression of LC3 protein stained as claybank region in the combined treatment group versus monotherapy (0.19±0.02 *vs*. 0.34±0.03, p<0.01) (Fig. 6C). DOX treatment resulted in a marked increment in TUNEL positive cells in comparison with vehicle and EGCG-treated cells. Moreover, co-treatment resulted in a higher rate compared with DOX treatment tumor (10.47±0.92 *vs*. 5.27±0.43, p<0.01) (Fig. 6D). Simultaneously, western blotting analysis of protein extracted from the tumor tissues showed a marked suppression in the expression of Atg5 and beclin1 (Figure not shown).

## Discussion

DOX, a representative agent of anthracyclines, has been world-widely in use for more than 40 years for the treatment of malignant neoplasm from various organs such as leukemia, breast cancer, colon cancer and hepatocellular carcinoma. However, the intrinsic shortage of DOX severely limits its clinical efficacy since minimizing dose may impair the therapeutic lethality and cause drug resistance, while dose escalation results in a dose-dependent cardiotoxicity [31], [32]. With this regard, it highlights an urgent need for combination of different chemotherapeutic agents mediated by potential complementary mechanisms, which severs as a common method to diminish the drug cytotoxicity and achieve more satisfied efficacy [33]. The present study revealed that combination of EGCG and DOX interacted to kill hepatocellular carcinoma cells via autophagy inhibition.

Autophagy, a lysosome-dependent degradation pathway which widely occurs in all eukaryotic cells [34], has been implicated in many physiological and pathological processes. Autophagy acts as a process which could result in both cell survival and cell death and its relationship with the HCC therapy has attracted increasing attention in a variety of fields in recent years. Interestingly, autophagy is inducible with the adoption of traditional chemotherapeutic drugs, including DNA-damaging agents, proteasome inhibitors and multikinase inhibitors resulted in impaired chemotherapeutic efficacy and the role for autophagy was believed to be a potential prosurvival and escapable mechanism [19], [35]–[38]. Recent findings have revealed that autophagy was a potential self-protection behavior responds to chemotherapeutic agents, which is a promising druggable target for anticancer therapy [39].

It is well known that DOX may initiate free radicals, generate reactive oxygen species (ROS) and then cause cytotoxicities via DNA damage [22]. Additionally, in this study, DOX was found to significantly trigger elevated LC3 fluorescent punctae and biochemical hallmarks of autophagy such as Atg5 and beclin1 in Hep3B cells, which indicated that autophagy signal was definitely involved in DOX-driving cytotoxicities process. To clarify whether autophagy plays a role in the mechanism underlying its antitumor effects or potential cancer elusion, we evaluated the influence of DOX on the cell viability by suppressing autophagy. As a result, compare with DOX alone, a more dramatic cell death and growth inhibition was found in DOX-treated settings with 3MA, an antagonist for autophagy. Similarly, results from a research have provided an evidence that autophagic pharmacologic inhibitors or autophagic genes disruption augments the proapoptotic activity for doxorubicin and melphalan, the DNA-damaging chemotherapeutic agents in human multiple myeloma cells [38]. Another study revealed that blocking autophagic flux also enhanced the lethality for sorafenib in HCC [19]. These data supported that DOX induced-autophagy was not responsible for its antitumor efficacy, but a potential self-protection behavior to escape from cytotoxicity therapy. Therefore, autophagy inhibition increases the sensitivity of HCC to DOX, which holds prospect for the potential application of autophagic inhibitors for cancer therapy. As the autophagic inhibitors, chloroquine and its analog, hydroxychloroquine have currently being evaluated in clinical trials for cancer therapy. However, chloroquine and hydroxychloroquine were reported to induce ocular toxicities, such as retinopathy [40]. And whether the safely tolerated doses of hydroxychloroquine or chloroquine exert effectively autophagic suppressing actions in human tumors has not yet been identified. These issues drew the need for additional novel inhibitors of autophagy without conspicuous observable toxicity [39].

Accumulative studies have revealed that many beneficial properties have been attributed to EGCG, including chemopreventive, anticarcinogenic, and antioxidant actions [41]. There has been studies suggested that EGCG induced autophagy, which resulted in decreased level of a mediator of lethal systemic inflammation, the high-mobility group protein B1 (HMGB1) [24]. Intriguingly, EGCG was also found to promisingly suppress autophagic level in varies cells with exception of its common pharmacological machinery [23], [25], [26]. Hashimoto, K. et al. [23] found that EGCG inhibited the formation of secondary lysosomes, autophagosomes and LC3-GFP in mouse-macrophage-like cell line. As for cancers, Zhang Y et al. [26] found that EGCG treatment dramatically blocked autophagic flux by elevating the conversion from LC3I to LC3II and increasing the accumulation of p62 in HepG2 cells, which was cell death-independent. This contrary actions performed by EGCG depends on cellular settings and different molecular pathways. However, the compromised autophagy responds to EGCG, is mostly adverse to most of chemotherapeutic agents in clinic. This study presented the interaction of autophagy and cell death following EGCG treatment. By using varies assays including electron microscopy, western blotting and qRT-PCR, EGCG was found to obviously down-regulate the basal autophagic activity in Hep3B cells with a dose-dependent pattern. Moreover, such autophagy inhibitory effect by EGCG was confirmed *in vivo* studies. In parallel, MTT assay indicated that EGCG dose-dependently exerted growth inhibition function in those cells. Thus, besides its anticancer effects, EGCG emerges as a novel therapeutic anticancer potential agent to substantially blunt autophagy signal in HCC.

Given a complementary mechanism responsible for antitumor effects between DOX and EGCG, it is worth determining the co-therapeutic effects by using these two compounds together. The present study indicated that combined treatment resulted in marked autophagy inhibition and cell death as well as apoptosis by both *in vivo* and *in vitro* binding assay, compared with monotherapy. Concomitantly, EGCG was found to abolish DOX-induced autophagy by western blot (data not shown). Given this amazing prospect, pharmacological and genetic approaches were used to modulate the activity of autophagy. As a consequence, blocking autophagy with Atg5 or beclin1 siRNAs resulted in greater cell death percentage, while activating autophagy with rapamycin resulted in impaired lethality in co-treatment Hep3B cells. This potently proved that inducible autophagy is the mechanism potentially underlying limited chemotherapeutic efficacy for DOX. Emerging studies have revealed that EGCG sensitize tumor cells to anticancer agents via different mechanisms. Prior study in multidrug resistance HCC confirmed that tea catechins at non-toxic doses(<100 µg/ml) augmented DOX-induced cell death and sensitize chemoresistant HCC cells to DOX via downregulaion of MDR1 expression, or enhancement of intracellular DOX accumulation, involving inhibition of P-gp function [7]. Study in orthotopic mouse glioblastoma models have shown that EGCG enhanced therapeutic efficacy of temozolomide, which is also a DNA damaging agent, through the inhibition of GRP78 [42]. Additionally, a sensitizing effect on doxorubicin-resistant murine sarcoma and human colon carcinoma cell lines was confirmed following combination of EGCG and DOX. Although several mechanisms underlying the anticancer activities of EGCG have been reported, the mechanism of EGCG action has not yet fully elucidated. The present study provided evidence that EGCG synergistically facilitated DOX anticancer efficacy via autophagy inhibition, a complementary mechanism between these compounds, which may be an effective targeting approach in cancer therapy.

It should reinforce the point that DOX-induced cardiotoxicity is a major limiting factor in anticancer therapy. Epidemiological studies have repeatedly demonstrated that health benefits a lot from green tea, and EGCG is the principal active constituent [43]. Specifically, EGCG was reported to protect heart against doxorubicin-induced myocyte injury [44]. Moreover, autophagy suppression mediated by resveratrol, also a plant-derived polyphenol, is an important mechanism to protect against DOX cardiotoxicity [45]. Hence, it is convinced that DOX combined with EGCG resulted in aggravated cytotoxicity to cancer cells and it seems plausible that this compound also acted protective role to myocardial cells.

The present research confirmed that a therapeutic regimen that EGCG co-treatment with DOX jointly exacerbated the antineoplastic efficacy mediated by suppressing autophagy. With regard to the unsatisfying efficacy for the therapy of liver cancer currently, the use of EGCG as a sensitizer for DOX chemotherapy warrants further clinical exploration. Compared with traditional anticancer agents, EGCG is available worldwide and safe to administrate in a wide range of dose. Promisingly, since exhibiting potential clinical benefits, EGCG has been strongly confirmed as a chemopreventive agent in clinical trials for varies cancers such as prostate, oral and colon cancer [46]–[49]. Given the evidence from these phase II and phase III clinical trials, “from bench to real-life situations” for EGCG as a superb agent for cancer chemoprevention is on the horizon. Indeed,there are still some problems remained to be answered. For instance, it is not clear that the particular mechanism of EGCG towards autophagy and specific signaling pathways network within the cooperated pattern in terms of cell survival and death. Secondly, the injurious dosage of EGCG has not yet been elucidated adequately, but the low dosage is preferred due to the safety concern. Although similar synergistic *in vitro* anti-cancer effect of EGCG and DOX was confirmed on HepG2 cells (Table S1), further *in vivo* study should be certified with other tumorigenic hepatoma cell lines. All together, this study demonstrated that combination of EGCG and DOX enhances the anticancer effects and targeting autophagy pathway might shed new light on improving the chemotherapeutic efficacy in HCC treatment.

## Supporting Information

Table S1 **Dosage inhibitory effects of both EGCG and DOX on the proliferation of HepG2 cells (n = 6).**
(DOCX)
