# Supplementary material for: Autophagy Inhibition Contributes to the Synergistic Interaction between EGCG and Doxorubicin to Kill the Hepatoma Hep3B Cells
Source: PLoS One. 2014 Jan 21;9(1):e85771. doi: 10.1371/journal.pone.0085771 (PMC3897495; doi:10.1371/journal.pone.0085771)
Supplement: Table S1 — Dosage inhibitory effects of both EGCG and DOX on the proliferation of HepG2 cells (n = 6). (DOCX) [file pone.0085771.s001.docx]

**Table S1. Dosage inhibitory effects of both EGCG and DOX on the proliferation of HepG2 cells (n=6)**

| EGCG  (µg/ml) | DOX  (µM) | Growth inhibitory effects(OD) | | | CDI |
| --- | --- | --- | --- | --- | --- |
|  |  | EGCG | DOX | EGCG+DOX |  |
| 0 | 0 | 0.94±0.05 | 0.94±0.05 | 0.94±0.05 |  |
| 5 | 0.0625 | 0.96±0.01 | 0.84±0.06 | 0.87±0.02 | 1.08±0.07 |
| 10 | 0.125 | 0.96±0.08 | 0.77±0.07 | 0.74±0.10 | 1.00±0.12 |
| 20 | 0.25 | 0.86±0.09 | 0.68±0.10 | 0.43±0.02 | 0.73±0.22 |
| 40 | 0.5 | 0.86±0.05 | 0.54±0.08 | 0.41±0.04 | 0.88±0.23 |
| 80 | 1 | 0.55±0.08 | 0.46±0.01 | 0.20±0.12 | 0.80±0.18 |

Drug interaction was measured as described in materials and methods with increasing concentrations of EGCG, DOX or both agents for 48h. CDI﹤1 indicates a synergistic effect, CDI=1 indicates an additive effect,CDI﹥1 indicates an antagonistic effect.
